# Use of laser microdissection for the construction of *Humulus japonicus* Siebold et Zuccarini, 1846 (Cannabaceae) sex chromosome-specific DNA library and cytogenetics analysis

**DOI:** 10.3897/CompCytogen.v8i4.8473

**Published:** 2014-12-10

**Authors:** Nickolay A. Yakovin, Mikhail G. Divashuk, Olga V. Razumova, Alexander A. Soloviev, Gennady I. Karlov

**Affiliations:** 1 Centre for Molecular Biotechnology, Russian State Agrarian University – Moscow Timiryazev Agricultural Academy, Moscow 127550, Timiryazevskaya street, 49, Russia; 2 Departament of Genetics, Biotechnology and Plant Breeding, Russian State Agrarian University – Moscow Timiryazev Agricultural Academy

**Keywords:** Laser microdissection, plant sex chromosomes, fluorescence in situ hybridization, chromosome-specific DNA

## Abstract

Dioecy is relatively rare among plant species, and distinguishable sex chromosomes have been reported in few dioecious species. The multiple sex chromosome system (XX/XY1Y2) of *Humulus japonicus* Siebold et Zuccarini, 1846 differs from that of other members of the family Cannabaceae, in which the XX/XY chromosome system is present. Sex chromosomes of *Humulus japonicus* were isolated from meiotic chromosome spreads of males by laser microdissection with the P.A.L.M. MicroLaser system. The chromosomal DNA was directly amplified by degenerate oligonucleotide primed polymerase chain reaction (DOP-PCR). Fast fluorescence *in situ* hybridization (FAST-FISH) using a labeled, chromosome-specific DOP-PCR product as a probe showed preferential hybridization to sex chromosomes. In addition, the DOP-PCR product was used to construct a short-insert, *Humulus japonicus* sex chromosomes-specific DNA library. The randomly sequenced clones showed that about 12% of them have significant homology to *Humulus lupulus* and 88% to *Cannabis sativa* Linnaeus, 1753 sequences from GenBank database. Forty-four percent of the sequences show homology to plant retroelements. It was concluded that laser microdissection is a useful tool for isolating the DNA of sex chromosomes of *Humulus japonicus* and for the construction of chromosome-specific DNA libraries for the study of the structure and evolution of sex chromosomes. The results provide the potential for identifying unique or sex chromosome-specific sequence elements in *Humulus japonicus* and could aid in the identification of sex chromosome-specific repeat and coding regions through chromosome isolation and genome complexity reduction.

## Introduction

Dioecy is relatively rare in the plant kingdom, in which only approximately 4% of angiosperm species are dioecious (Yampolsky and Yampolsky 1922). Most of these species lack morphologically distinguishable sex chromosomes and posses sex-determining loci on homologous chromosomes or utilize environmental cues to determine sex ratios (Ainsworth 2000, Charlesworth and Guttman 1999, Tanurdzic and Banks 2004). Distinguishable sex chromosomes have been reported in several dioecious species belonging to five angiosperm families. One of these, *Humulus japonicus* Siebold et Zuccarini, 1846 (Japanese hop), is a dioecious species of the family Cannabaceae. The chromosome number is 2n=16=14+XX for females and 2n=17=14+XY1Y2 for males (Winge 1929). The multiple sex chromosome system (XX/XY1Y2) of *Humulus japonicus* differs from other members of the family Cannabaceae, such as the common hop (*Humulus lupulus* Linnaeus, 1753, 2n=20) and hemp (*Cannabis sativa* Linnaeus, 1753, 2n=20), in which the XX/XY chromosome system is present. Additionally, the genome sizes of these three related species vary widely: *Humulus lupulus* – 2.90 pg (Zonneveld et al. 2005), *Humulus japonicus* – 1.7 pg (Grabowska-Joachimiak et al. 2006) and *Cannabis sativa* - 0.9 pg (Bennett and Leitch 2010; Sakamoto et al. 1998). Therefore, the family Cannabaceae can be used as a model to study the evolution of plant sex chromosomes in addition to plants from the genera *Silene* Linnaeus, 1753 and *Rumex* Linnaeus, 1753, which are classically used in this regard. In spite of recent progress in the *H. lupulus, H. japonicus* and *Cannabis sativa* molecular cytogenetics (Alexandrov et al. 2012; Divashuk et al. 2011, 2014; Grabowska-Joachimiak et al. 2011; Karlov et al. 2003; Kim et al. 2008;) and *Cannabis sativa* genomics (van Bakel et al. 2012), we know little about the genetics of sex determination in these species (Ming et al. 2011).

The most widespread method for the detection of new sex-specific DNA sites is to search for molecular markers that are linked to sex (Alexandrov et al. 2011; Danilova and Karlov 2006; Gao et al. 2010; Polley et al. 1997), but this method does not allow for the study of multiple chromosome-specific sequences. In complex plant genomes containing widespread repetitive sequences, it is important to establish genomic resources that enable us to focus on a particular part of the genome. There are several methods available that can be used to dissect a particular chromosome or subchromosomal region. The direct strategy for isolating sequences from chromosomes of interest is to separate them by a flow-sorting procedure or by microdissection. The main disadvantage of the flow-sorting approach is contamination of dissected material by chromosomes of similar size and the presence of particles with the same DNA content as sorted chromosomes (Dolezel et al. 2001). Currently, microdissection constitutes one of the most direct approaches to ascertain the molecular composition of certain chromosomes or chromosome regions (Houben 2012). Fine glass needles are commonly used for the mechanical dissection of chromosomes. Alternatively, laser microdissection results in the isolation of extremely pure pools of chromosomes, from which DNA can be amplified by DOP-PCR (degenerate oligonucleotide primed PCR) both to generate chromosome-specific DNA libraries and to be applied as complex probes for FISH (Fukui et al. 1992; Hobza et al. 2004; Houben 2012).

In plants, Sandery et al. (1991) first applied the microdissection technique toward isolating B-chromosomes from rye (*Secale cereale* Linnaeus, 1753) and were able to identify a DNA sequence on these rye B-chromosomes. With the development of PCR, microdissection techniques have widely been used with genetic studies of *Secale cereale* (Houben et al. 1996; Zhou et al. 1999), *Triticum aestivum* Linnaeus, 1753 (Hu et al. 2004), *Zea mays* Linnaeus, 1753 (Stein et al. 1998), *Avena sativa* Linnaeus, 1753 (Chen and Armstrong 1995; Sanz et al. 2012), *Gossypium arboreum* Linnaeus, 1753 (Renhai et al. 2012), *Citrus grandis* Osbeck, 1757 (Huang et al. 2004a,b), *Silene latifolia* Poiret, 1789 (Hobza et al. 2004, 2007), *Populus tremula* Linnaeus, 1753 (Zhang et al. 2005), an addition line of wheat-*Thinopyrum intermedium* Barkworth & Dewey, 1985 (Deng et al. 2013a) and *Spinacia oleracea* Linnaeus, 1753 (Deng et al. 2013b). Chromosome microdissection and cloning are powerful tools that combine cytogenetics with molecular genetics and have played an important role in research on genome structure (Fominaya et al. 2005; Hobza and Vyskot 2007). By generating a DNA probe for fluorescent *in situ* hybridization (FISH) with the DNA microdissected from a certain chromosome, it is possible to obtain an idea of the DNA sequences shared among different chromosomes within the same genome. The microdissection technique was used to study the structure and evolution of sex chromosomes from two model species, *Rumex acetosa* and *Silene latifolia* (Mariotti et al. 2006, Matsunaga et al. 1996, 1999; Shibata et al. 1999). These species possess heteromorphic sex chromosomes that can be microscopically distinguished from the remaining complement chromosomes (Vyskot and Hobza 2004). Painting of sex chromosomes has been performed in *Rumex acetosa* Linnaeus, 1753 by Shibata et al. (1999) and in *Silene latifolia* by Hobza et al. (2004). Hobza et al. (2004) used a modified FAST-FISH protocol, based on a short hybridization time combined with a low concentration of probe, and successfully distinguished the sex chromosomes by differential labeling patterns.

Identification of specific chromosomes for microdissection is difficult in many plant species. It can be achieved by choosing a plant with chromosomes bearing a prominent morphological feature, for example, a large somatic chromosome such as the Y chromosome in *Silene*. In *Humulus japonicus*, sex chromosomes are difficult to distinguish from autosomes at the mitotic metaphase fig (Grabowska-Joachimiak et al. 2011; Kim et al. 2008). During meiosis in the male plants of *Humulus japonicus*, a trivalent chromosome configuration is observed (Jacobsen 1957). This can be most clearly observed at diakinesis and metaphase I, which allows for reliable identification of sex chromosomes from autosomes in pollen mother cells (PMC). PMC at these stages of meiosis can easily be isolated in large quantities from immature male flowers.

To investigate the structure of the sex chromosomes in *Humulus japonicus*, the XY1Y2 chromosomes were isolated by laser microdissection of the meiotic trivalent at the diakinesis and metaphase I stages and the DOP-PCR products were used for FISH and the creation of the DNA library.

## Materials and methods

### Plant material and chromosome preparation

The male *Humulus japonicus* plants (2n=17=14+XY1Y2) were grown in a greenhouse from seeds of cultivar “Samuray” (“Gavrish seeds”, Moscow, Russia) and were used to prepare the meiotic chromosomes. The one month old plants were exposed to a short day photoperiod (8 h day and 16 h night) to induce flowering.

For the preparation of *Humulus japonicus* meiotic diakinesis and metaphase I chromosomes, the significantly modified method of Zhong et al. (1996) was used. Young floral buds from male plants, approximately 3~5 mm long, were selected for meiotic chromosome preparation and the appropriate meiotic stage of development was determined. One anther from a bud was squashed in 1% Carmine in 45% acetic acid on a slide and observed under a phase microscope. The remaining anthers with pollen mother cells (PMCs) in metaphase I were fixed in a mixture of glacial acetic acid and absolute ethanol (1:3) for 1 h, washed twice on the surface of distilled water in a Petri dish (5 cm in diameter) and placed on 50 µmol L^-1^ citrate buffer (pH 4.5) for 10 minutes. Digestion was carried out on the surface of an enzyme mixture containing 3 % (w/v) cellulase R-10 (Sigma), 0.3% (w/v) pectinase (Sigma) and 0.3 % (w/v) cytohelicase (Sigma). A cell spreading technique was used for meiotic chromosome preparation on microscope slides covered with a polyethylene naphthalate membrane (P.A.L.M. GmbH, Bernried, Germany), and the slides were used for microdissection.

For FISH experiments, the chromosome preparations were made as described above, except that conventional slides were used instead of the polyethylene naphthalate membrane-coated slides.

### Microdissection

The P.A.L.M. MicroLaser system (P.A.L.M. GmbH) was used to dissect Y1-X-Y2 trivalent figures at diakinesis. The microscopic stage, micro-manipulator and laser micromanipulation procedures were computer controlled. All procedures for the dissection of chromosomes are adapted from experiments performed by Kubickova et al. (2002). The membrane around the chromosome of interest is cut, and the chromosome is then catapulted by a single laser pulse directly into the cap of an Eppendorf tube. Energy of 1.5–11.7 mJ per pulse is used for microdissection and 2 mJ per pulse is used for catapulting. Fifty trivalents were collected in each experiment. The isolated chromosomes were collected in 20 μl of distilled water in a tube.

### DOP-PCR

Chromosomes were used directly (without any enzymatic treatment) for amplification by DOP-PCR with regular primers designed by Telenius et al. (1992). Amplification reactions containing 50 isolated sex chromosomes were brought to volumes of 25 μL containing final concentrations of 1 x Taq DNA polymerase buffer, 0.2 mM each of four deoxynucleotides, 1.5 pM DOP primer and 0.02 U/μL Taq DNA polymerase. Amplifications were performed in a Tetrad PCR machine. An initial incubation of 94°C for 4 min was followed by eight thermal cycles of 94°C for 1 min, 28°C for 1 min, and 72°C for 2 min, in which the duration of the heating step between 28 and 72°C was set to 2 min. This was followed by 30 cycles of 94°C for 1 min, 50°C for 1 min, and 72°C for 2 min, with a single final incubation at 72°C for 7 min.

A male-speciﬁc DNA marker (Gao et al. 2010) was used to check the quality of DOP-PCR product from sex chromosomes. The PCR was performed using primers Sex164F 5’- AGAGAGAGAGAGAGAGCGAGAAAG-3’ and Sex164R 5’-AGAGAGAGAGAGAGAGCGGAAATG-3’. Ampliﬁcation reactions were performed using a Tetrad PCR machine after initial incubation at 94 C for 4 min, which was followed by 30 cycles at 94°C for 30 s, 60°C for 45 s, and 72 C for 45 s, with a single final incubation at 72°C for 7 min.

### DOP-PCR product labeling and FISH

For FISH experiments, the DOP-PCR products were labeled with dioxigenin-11-dUTP (Roche Diagnostics GmbH). One-half of a microliter of the primary PCR reaction was added as a temfig to 20 µl of DOP-labeling PCR mix. Cycling parameters were: 3 min at 95°C for initial denaturation; 30 cycles of 15 s at 94°C, 30 s at 56°C; and 2 min at 72°C, followed by a 5 min final extension at 72°C.

FISH was performed using a modified version of the method of Fransz et al. (1996). The slides were preheated at 60^o^C for 30 min, pretreated with 100 μg mL^-1^ DNase-free RNase in 2 x SSC at 37^ o^ C for 1 h and then washed three times in 1xPBS for five minutes each. 30 μL of hybridization mixture containing 50% formamide, 2x SSC, 10% sodium dextran sulphate, 50 mmol L^-1^ phosphate buffer (pH 7.0) and 10-20 ng µL^-1^ of DNA probe was used for each slide. *In situ* hybridization was performed at 37°C overnight, followed by post-hybridization wash for 15 minutes in 0.1x SSC at 42°C.

The FAST-FISH was performed as described by Hobza et al. (2004). The pretreatment and hybridization mixture preparation for the slides was as described above. The time of *in situ* hybridization was shortened to 1 h.

The slides were counterstained with 4,6-diamino-2-phenylindole (DAPI, 0,5 µg/ml) in Vectashild (Vector). The hybridization signals were observed under a fluorescence microscope (Zeiss AxioImager.M1, Germany). Images were captured by a charge-coupled device (CCD) system (AxioCam MRm) and AXIOVISION software.

### Library preparation and sequencing

The DOP-PCR products were cloned into the pGEM®-T Easy Vector System (Promega, USA) as described by manufacturer. Clones were picked into 96 well figs, grown for 18 h, replicated and frozen at -80° C. One hundred randomly selected clones were tested by PCR with M13 primers on the insert present, and 24 randomly selected clones were sequenced using ABI Big Dye Mix v3.1 (Applied Biosystems Inc) with M13 primers, according to the manufacturer’s instructions. Products were resolved on an ABI 3130xl sequencer. BLAST analysis was performed according to the standard procedure. BLAT analysis was used to find homology of sequences against the *Cannabis sativa* genome (http://genome.ccbr.utoronto.ca/index.html). BLAT on DNA is designed to quickly find sequences of 95% and greater similarity of length 25 bases or more.

## Results

**Figure 1.**
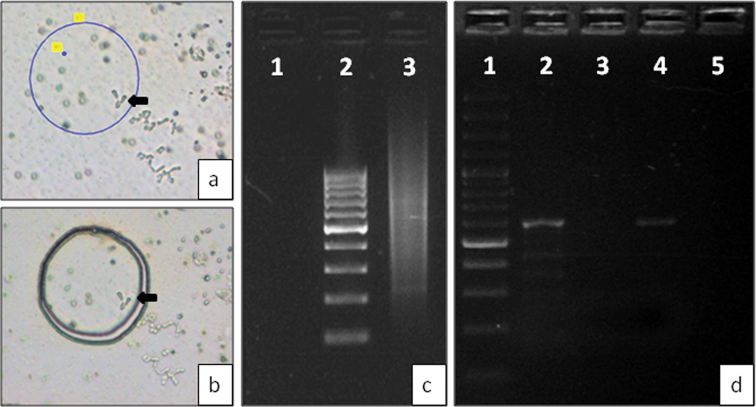
Microdissection of *Humulus japonicus* sex chromosomes at meiotic diakinesis-metaphase I stage. **a** Selection of sex chromosomes (Y1-X-Y2 trivalent formation indicated by arrow) **b** Cutting out of the sex chromosomes **c** The gel electrophoresis of the microdissected sex chromosomes DOP-PCR product: 1 – negative control, 2 – 100 bp DNA ladder, 3 - DOP-PCR **d** The gel electrophoresis after PCR with the male sex specific marker on different DNA temfigs: 1 – 100 bp DNA ladder, 2 – DOP-PCR product from sex chromosomes, 3 – DOP-PCR product from autosomes, 4 – DNA of male plant, 5 – DNA of female plant.

The sex chromosomes from PMCs at meiotic diakinesis and metaphase I stages of *Humulus japonicus* can easily be distinguished from autosomes under a light microscope without any staining procedures, which allows for reliable identification and rapid isolation of pure chromosomes of interest (Fig. 1a). The sex chromosomes were bordered and cut using a laser beam of low energy, transferred by a single laser pulse directly into the cap of an Eppendorf tube (Fig. 1b) and then directly (without any enzymatic treatment) used as temfig for DNA amplification. This procedure minimizes the level of contamination. On one slide, we were able to collect up to approximately 50 sex trivalents (Y1-X-Y2). After amplification by DOP-PCR, agarose gel electrophoresis showed that DNA fragments varied in size from approximately 200 bp to 3000 bp. The conditions of DOP-PCR were optimized to minimize any preferential amplification (Fig. 1c). The absence of banding on the gel indicates preferential amplification.

To ensure that DOP-PCR product was obtained from sex chromosomes the male specific SCAR marker was used. The PCR product of expected size was obtained from DOP-PCR DNA and DNA from male plants only. No amplification was detected from female DNA and DOP-PCR product obtained after microdissection of autosomes (Fig. 1d), indicating no cross contamination.

To examine the quality of the DOP-PCR product, the standard FISH procedure was performed. DIG-labeled DOP-PCR products hybridized to the chromosomes of male plants in the absence of a competitor. Signals were observed uniformly on all chromosomes (data not shown).

**Figure 2.**
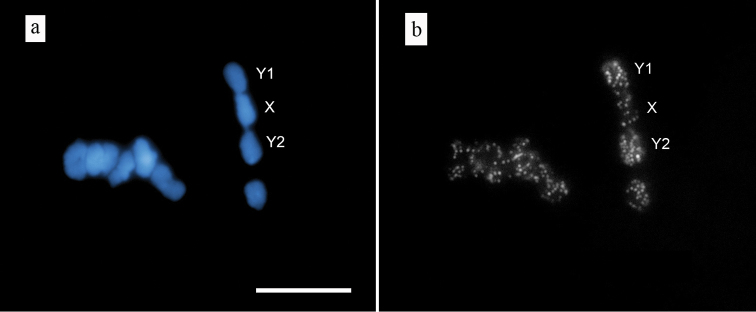
FISH with DOP-PCR probe on meiotic chromosomes of *Humulus japonicus*. **a** DAPI-stained chromosomes at meiotic metaphase I stage **b** The result of FAST-FISH with DOP-PCR probe. The Y1-X-Y2 trivalent formation is indicated. Bar = 10 µm.

The application of FAST-FISH, using lower concentrations of DIG-labeled DOP-PCR probe per slide and reducing the hybridization time from 16 h to 1 h, allowed for the differentiation of chromosomes by FISH signal (Fig. 2). Analysis of the 25 meiotic metaphase I chromosome figs shows that the intensity of FISH signal on the Y1 and Y2 chromosomes was higher compared to chromosome X and autosomes.

The DOP-PCR product was used to construct a short-insert *Humulus japonicus* sex chromosomes-specific DNA library. Cloning of the DOP-PCR products resulted in 5 x 10^3^ recombinant colonies per 100 µl PCR reaction mixture. The length of the cloned DNA fragments ranged from 450 to 3000 bp, with an average fragment length of 1000 bp. Twenty-four clones were randomly selected for sequencing. When we compared sequences with the NCBI database, using BLAST, 11 of them showed homology to sequences of plant retrotransposons (Table 1).

Three sequences show homology to some sequences of *Humulus lupulus* and 13 sequences show homology to *Cannabis sativa*. Two sequences show homology to hypothetical proteins or mRNA. Additionally, a database search of the recently sequenced *Cannabis sativa* [14] using BLAT (http://genome.ccbr.utoronto.ca) showed homology in 21 of 24 sequences with the *Cannabis* genome (Table 1).

**Table 1.** Similarity of the sequenced *Humulus japonicus* sex chromosome specific clones to GenBank accessions, *Cannabis sativa* draft genome and RepBase database.

№	Similarity to GenBank accessions	Tool	Similarity to *Cannabis sativa* ***
1	*Humulus lupulus* clone HlAT9 microsatellite sequence (AY588370.1)	blastn *	+
	gag-pol polyprotein [*Phaseolus vulgaris*] (AAR13317.1)	blastx *	
2	*Medicago truncatula* DNA sequence from clone MTH2-46C14 on chromosome 3, complete sequence (CT962505.9)	blastn	+
	pol protein [Cucumis melo subsp. melo] (AAO45752.1)	blastx	
3	No homology in GenBank and RepBase		+
4	*Medicago truncatula* chromosome 5 clone mte1-70c24, COMPLETE SEQUENCE (CR932962.2)	blastn	+
5	retrotransposon gag protein [Cucumis melo subsp. melo] (ADN33993.1)	blastn	+
	integrase [*Populus trichocarpa*] (ABG37658.1)	blastn	
6	*Populus trichocarpa* clone POP065-M23, complete sequence (AC209187.1)	blastn	+
	pol protein [Cucumis melo subsp. melo] (AAO45752.1)	blastx	
	rve superfamily: Integrase core domain (pfam00665)	blastx	
7	No homology in GenBank and RepBase		+
8	*Serratia proteamaculans* 568, complete genome (CP000826.1)	blastn	-
9	No homology in GenBank and RepBase		-
10	*Nicotiana benthamiana* mRNA for PME inhibitor (FN432042.1)	blastn	+
11	A family of autonomous Polinton DNA transposons (CR1-6_BF)	CENSOR **	+
12	*Gossypium raimondii* clone GR__Ba0005I14-jfn, complete sequence (AC243106.1)	blastn	+
	Amphioxus CR1-6_BF autonomous Non-LTR Retrotransposon - consensus.	CENSOR	
13	*Lotus japonicus* cDNA, clone: LjFL1-045-CB01, HTC (AK337120.1)	blastn	+
	integrase [Populus trichocarpa] (ABG37658.1)	blastx	
	LTR retrotransposon from the western balsam poplar: internal portion. (Gypsy-39_PT-I)	CENSOR	
14	No homology in GenBank and RepBase		-
15	*Humulus lupulus* vps gene for valerophenone synthase, complete cds (AB047593.2)	tblastx *	+
	gag-pol polymerase [Arabidopsis lyrata subsp. lyrata] (ABW81018.1)	blastx	
16	gag-protease polyprotein [Cucumis melo subsp. melo] (AAO45751.1)	blastx	+
17	hypothetical protein VITISV_026408 [*Vitis vinifera*] (CAN60970.1)	blastx	+
18	*Humulus lupulus* clone GT2-P16-8 microsatellite sequence (EU094990.1)	blastn	+
	HLUTR3CH_T3_051_H10_24JUL2006_066 HLUTR3CH *Humulus lupulus* cDNA, mRNA sequence (GD252950.1)	blastn	
19	*Cannabis sativa* strain Purple Kush scaffold130939_1, whole genome shotgun sequence (AGQN01284755.1)	blastn (wgs)	+
20	No homology in GenBank and RepBase		+
21	gag-protease polyprotein [Cucumis melo subsp. melo] (AAO45751.1)	blastx	+
	*Vitis vinifera* contig VV78X146750.38, whole genome shotgun sequence (AM458430.2)	tblastx	
22	No homology in GenBank and RepBase		+
23	No homology in GenBank and RepBase		+
24	*Daucus carota subsp. sativus* clone BAC C235O6O genomic sequence (FJ148580.1)	blastn	+
	Retrotransposon gag protein [*Asparagus officinalis*] (ABD63156.1)	blastx	

* GenBank database<br/> ** RepBase database (http://www.girinst.org/censor/index.php)<br/> *** Seach with BLAT tool in *Cannabis sativa* genome (http://genome.ccbr.utoronto.ca)

## Discussion

To isolate sex chromosomes, we used a technique based on laser beam microdissection with the P.A.L.M. MicroLaser system. An accurate identification of the target chromosomes is the first step in microdissection and microcloning. Additionally, the quality of microdissected chromosomal DNA depends critically on the pretreatment, chromosome fixation and staining of the samples (Houben 2012). On mitotic metaphase figs, the sex chromosomes of *Humulus japonicus* are difficult to distinguish from autosomes without special staining procedures. C-banding/DAPI or FISH with subtelomeric repeat were proposed to identify the X-, Y1-and Y2-chromosomes (Alexandrov et al. 2012; Grabowska-Joachimiak et al. 2011). Pretreatment and UV-light can damage chromosomal DNA when using these methods (Houben 2012). In our study, the chromosomes from PMCs at meiotic diakinesis -and metaphase I stages were used. At these stages, the sex chromosomes of *Humulus japonicus* (trivalent chromosome configuration) can easily be distinguished from autosomes under a light microscope without any staining procedures, which allows for reliable identification and rapid isolation of pure chromosomes of interest. Sufficient dispersion of chromosomes suitable for laser microdissection was achieved by spreading procedure of PMCs on microscopic slides covered with a polyethylene naphthalate membrane. Another advantage of the use of PMCs is the high level of synchronization of the cells.

The results of standard FISH procedure with DIG-labeled DOP-PCR products is in agreement with previous observations showing that the DNA of microdissected plant chromosomes hybridized to all chromosomes as a result of widespread repetitive sequences contained in plant genomes (Hobza et al. 2004). The use of complex subgenomic probes often leads to a nonspecific FISH signal on all chromosomes due to the difference in complexity of genomes and organization of repetitive sequences in plants compared to animals (Heslop-Harrison and Schwarzacher 2011; Schmidt and Heslop-Harrison 1998; Schubert et al. 2001).

The preferential, uneven distribution of DOP-PCR probes on the Y1 and Y2 sex chromosomes in FAST-FISH experiments is indicative of an abundance of dispersed repeats, such as retrotransposons, on Y chromosomes. These results agree with Grabowska-Joachimiak et al. (2011) where DAPI/C-banding shows brighter staining of the Y1 and Y2 chromosomes. Additionally, it may indicate accumulation on Y chromosomes-specific repetitive DNA. The accumulation of different repetitive DNA sequences was detected on Y chromosomes of *Rumex* and *Silene* species (Hobza et al. 2006; Kejnovsky et al. 2009; Shibata et al. 1999; Steflova et al. 2013).

The observation that about 12% of the sequences show significant homology to *Humulus lupulus* and 88% to *Cannabis sativa*, whose genome is closely related to *Humulus japonicus*, indicates efficient amplification of DNA from *Humulus japonicus* chromosomes by DOP-PCR. Less apparent homology between *Humulus japonicus* and *Humulus lupulus*, compared to *Cannabis sativa*, can be explained by the lack of sequence representation in the GenBank database. FISH with DOP-PCR probes led to a hybridization signal on all chromosomes, which suggests that a large amount of dispersed repeated DNA sequences are present in the genome of this species and in the DOP-PCR product. This was confirmed by sequencing, which showed that 44% of sequences were homologous to plant retroelements. The presence of multiple sequences with homology to plant retrotransposons is in agreement with FISH experiments in which a dispersed signal was seen on all chromosomes, given that retroelements are usually distributed throughout the genomes of plants (Heslop-Harrison and Schwarzacher 2011). The preferential hybridization to Y chromosomes of sex chromosome-specific DOP-PCR probes in FAST-FISH experiments indicates the presence of chromosome-specific repeated sequences.

It was concluded that laser microdissection is a useful tool for isolating the DNA of individual chromosomes, including the relatively small chromosomes of *Humulus japonicus*, and for the construction of chromosome-specific libraries for the study of the structure and evolution of the sex chromosomes. This is the first time a DNA library of the sex chromosomes Japanese hop has been constructed.

## References

[B1] Ainsworth C (2000) Boys and girls come out to play: The molecular biology of dioecious plants. Annals of Botany 86: 211–221. doi: 10.1006/anbo.2000.1201

[B2] Alexandrov OS Divashuk MG Karlov GI (2011) Development of sex specific DNA marker for Japanese hop *Humulus japonicus* Siebold & Zucc. Russian Journal of Genetics 47: 1016–1020. doi: 10.1134/S1022795411080023 21954626

[B3] Alexandrov O Divashuk M Yakovin N Karlov G (2012) Sex chromosome differentiation in *Humulus japonicus* Siebold & Zuccarini, 1846 (Cannabaceae) revealed by fluorescence in situ hybridization of subtelomeric repeat. Comparative Cytogenetics 6: 239–247. doi: 10.3897/compcytogen.v6i3.3261 24260665 10.3897/CompCytogen.v6i3.3261 PMC3833800

[B4] Bennett MD Leitch IJ (2010) Angiosperm DNA *C*-values database (release 7.0, Dec. 2010). http://www.kew.org/cvalues/

[B5] Charlesworth D Guttman DS (1999) The evolution of dioecy and plant sex chromosome systems. In: Ainsworth C (Ed.) Sex Determination in Plants.BIOS Scientific Publishers, Oxford, 25–49.

[B6] Chen QF Armstrong K (1995) Characterization of a library from single microdissected oat (*Avena sativa* L.) chromosome. Genome 38: 706–714. doi: 10.1139/g95-089 18470198 10.1139/g95-089

[B7] Danilova TV Karlov GI (2006) Application of inter simple sequence repeat (ISSR) polymorphism for detection of sex-specific molecular markers in hop (*Humulus lupulus* L.). Euphytica 151: 15–21. doi: 10.1007/s10681-005-9020-4

[B8] Deng C Bai L Fu S Yin W Zhang Y Chen Y Wang RR-C Zhang X Han F Hu Z (2013a) Microdissection and chromosome painting of the alien chromosome in an addition line of wheat-*Thinopyrum intermedium*. PloS ONE 8(8): e72564. doi: 10.1371/journal.pone.0072564 23967319 10.1371/journal.pone.0072564 PMC3743814

[B9] Deng CL Qin RY Cao Y Gao J Li SF Gao WJ Lu LD (2013b) Microdissection and painting of the Y chromosome in spinach (*Spinacia oleracea*). Journal of Plant Research 126(4): 549–556. doi: 10.1007/s10265-013-0549-3 23381038 10.1007/s10265-013-0549-3

[B10] Divashuk MG Alexandrov OS Kroupin PY Karlov GI (2011) Molecular cytogenetic mapping of *Humulus lupulus* sex chromosomes. Cytogenetic and Genome Research 134: 213–219. doi: 10.1159/000328831 21709414 10.1159/000328831

[B11] Divashuk MG Alexandrov OS Razumova OV Kirov IV Karlov GI (2014) Molecular Cytogenetic Characterization of the Dioecious *Cannabis sativa* with an XY Chromosome Sex Determination System. PLoS ONE 9(1): e85118. doi: 10.1371/journal.pone.0085118 24465491 10.1371/journal.pone.0085118 PMC3897423

[B12] Dolezel J Lysak MA Kubalakova M Simkova H Macas J Lucretti S (2001) Sorting of plant chromosomes. Methods in Cell Biology 64(part B): 3–31. 11070830 10.1016/s0091-679x(01)64004-4

[B13] Fominaya A Linares C Loarce Y Ferrer E (2005) Microdissection and microcloning of plant chromosomes. Cytogenetic and Genome Research 109: 8–14. doi: 10.1159/000082376 15753553 10.1159/000082376

[B14] Fransz PF Stam M Montijn B Hoopen RT Wiegant J Kooter JM Oud O Nanninga N (1996) Detection of single-copy genes and chromosome rearrangements in *Petunia hybrida* by fluorescence in situ hybridization. Plant Journal 9: 767–774. doi: 10.1046/j.1365-313X.1996.9050767.x

[B15] Fukui K Minezawa M Kamisugi Y Ishikawa M Ohmido N Yanagisawa T Fujishita M Sakai F (1992) Microdissection of plant chromosomes by argon-ion laser beam. Theoretical Applied Genetics, 84: 787–791. 24201475 10.1007/BF00227385

[B16] Gao WJ Sha T Ji YK Deng CL Lu LD (2010) Clone and development of ISSR and SCAR markers linked to male *Humulus scandens*. Journal of Tropical and Subtropical Botany 18: 283–287.

[B17] Grabowska-Joachimiak A Mosiolek M Lech A Goralski G (2011) C-Banding/DAPI and in situ hybridization reflect karyotype structure and sex chromosome differentiation in *Humulus japonicus* Siebold & Zucc. Cytogenetic and Genome Research 132: 203–211. doi: 10.1159/000321584 21079383 10.1159/000321584

[B18] Grabowska-Joachimiak A Sliwinska E Pigula M Joachimiak A (2006) Genome size in *Humulus lupulus* L. and *H. japonicus* Siebold & Zucc. (Cannabaceae). Acta Societatis Botanicorum Poloniae 75: 207–214. doi: 10.5586/asbp.2006.024

[B19] Heslop-Harrison JS Schwarzacher T (2011) Organization of the plant genome in chromosomes. Plant Journal 66: 18–33. doi: 10.1111/j.1365-313X.2011.04544.x 21443620 10.1111/j.1365-313X.2011.04544.x

[B20] Hobza R Kejnovsky E Vyskot B Widmer A (2007) The role of chromosomal rearrangements in the evolution of *Silene latifolia* sex chromosomes. Molecular Genetics and Genomics 278: 633–638. doi: 10.1007/s00438-007-0279-0 17671795 10.1007/s00438-007-0279-0

[B21] Hobza R Lengerova M Cernohorska H Rubes J Vyskot B (2004) Chromosome Research 12: 245–250. doi: 10.1023/B:CHRO.0000021929.97208.1c 15125638 10.1023/b:chro.0000021929.97208.1c

[B22] Hobza R Lengerova M Svoboda J Kubekova H Kejnovsky E Vyskot B (2006) An accumulation of tandem DNA repeats on the Y chromosome in *Silene latifolia* during early stages of sex chromosome evolution. Chromosoma 115: 376–382. doi: 10.1007/s00412-006-0065-5 16612641 10.1007/s00412-006-0065-5

[B23] Hobza R Vyskot B (2007) Laser microdissection-based analysis of plant sex chromosomes. Methods in Cell Biology 82: 433–453. doi: 10.1016/S0091-679X(06)82015-7 17586267 10.1016/S0091-679X(06)82015-7

[B24] Houben A (2012) Chromosome microdissection and utilization of microisolated DNA. In: Bass HW Birchler JA (Eds) Plant Cytogenetics.Springer, New York, 257–270.

[B25] Houben A Kynast RG Heim U Hermann H Jones RN Forster JW (1996) Molecular cytogenetic characterization of the terminal heterochromatic segment of the B-chromosome of rye (*Secale cereale*). Chromosoma 105: 97–103. doi: 10.1007/BF02509519 8753699 10.1007/BF02509519

[B26] Hu ZM Wang H Shi R Dang BY Hu J Yin WB Chen YH Jung SM Chen ZH (2004) Microdissection and construction of region-specific DNA libraries of wheat chromosome 6B. Acta Botanica Sinica 46: 1357–1365.

[B27] Huang D Wu W Lu L (2004a) Microdissection and molecular manipulation of single chromosomes in woody fruit trees with small chromosomes using pomelo (*Citrus grandis*) as a model. II. Cloning of resistance gene analogs from single chromosomes. Theoretical and Applied Genetics 108: 1371–1377. doi: 10.1007/s00122-003-1562-z 14727033 10.1007/s00122-003-1562-z

[B28] Huang D Wu W Zhou Y Hu Z Lu L (2004b) Microdissection and molecular manipulation of single chromosomes in woody fruit trees with small chromosomes using pomelo (*Citrus grandis*) as a model. I. Construction of single chromosomal DNA libraries. Theoretical and Applied Genetics 108: 1366–1370. doi: 10.1007/s00122-003-1550-3 14727028 10.1007/s00122-003-1550-3

[B29] Jacobsen P (1957) Hereditas 43: 357–370. doi: 10.1111/j.1601-5223.1957.tb03444.x

[B30] Karlov GI Danilova TV Horlemann C Weber G (2003) Molecular cytogenetics in hop (*Humulus lupulus* L.) and identification of sex chromosomes by DAPI-banding. Euphytica 132: 185–190. doi: 10.1023/A:1024646818324

[B31] Kejnovsky E Hobza R Cermak T Kubat Z Vyskot B (2009) The role of repetitive DNA in structure and evolution of sex chromosomes in plants. Heredity 102: 533–541. doi: 10.1038/hdy.2009.17 19277056 10.1038/hdy.2009.17

[B32] Kim S-Y Kim C-S Lee J Bang J-W (2008) Karyotype analysis and physical mapping using two rRNA genes in dioecious plant, *Humulus japonicus* Siebold & Zucc. Genes and Genomics 30: 243–251.

[B33] Kubickova S Cernohorska H Musilova P Rubes J (2002) The use of laser microdissection for the preparation of chromosome-specific painting probes in farm animals. Chromosome Research 10: 571–577. doi: 10.1023/A:1020914702767 12498346 10.1023/a:1020914702767

[B34] Mariotti B Navajas-Pérez R Lozano R Parker JS de la Herrán R Rejón CR Rejón MR Garrido-Ramos M Jamilena M (2006) Cloning and characterization of dispersed repetitive DNA derived from microdissected sex chromosomes of *Rumex acetosa*. Genome 49: 114–121. 16498461 10.1139/g05-089

[B35] Matsunaga S Kawano S Michimoto T Higashiyama T Nakao S Sakai A Kuroiwa T (1999) Semi-automatic laser beam micro dissection of the Y chromosome and analysis of Y chromosome DNA in a dioecious plant, *Silene latifolia*. Plant Cell Physiology 40: 60–68. 10189703 10.1093/oxfordjournals.pcp.a029475

[B36] Matsunaga S Kawano S Takano H Uchida H Sakai A Kuroiwa T (1996) Isolation and developmental expression of male reproductive organ-speciﬁc genes in a dioecious campion, Melandrium album (*Silene latifolia*). Plant Journal 10: 679–689. doi: 10.1046/j.1365-313X.1996.10040679.x 8893544 10.1046/j.1365-313x.1996.10040679.x

[B37] Ming R Bendahmane A Renner SS (2011) Sex chromosomes in land plants. Annual Review of Plant Biology 62(1): 485–514. doi: 10.1146/annurev-arplant-042110-103914 10.1146/annurev-arplant-042110-103914 21526970

[B38] Polley A Ganal WM Seigner E (1997) Identification of sex in hop (*Humulus lupulus*) using molecular markers. Genome 40: 357–361. doi: 10.1139/g97-048 18464833 10.1139/g97-048

[B39] Renhai P Fang L Xiao H Chunying W Shaohui L Xiangdi Z Yuhong W Kunbo W (2012) Microdissection and microcloning of chromosome 5 in *Gossypium arboreum*. Plant Molecular Biology Reporter 30: 1218–1228. doi: 10.1007/s11105-012-0438-2

[B40] Sakamoto K Akiyama Y Fukui K Kamada H Satoh S (1998) Characterization: genome sizes and morphology of sex chromosomes in Hemp (*Cannabis sativa* L.). Cytologia 63: 459–464. doi: 10.1508/cytologia.63.459

[B41] Sandery MJ Forster JW Macadam SR Blunden R Jones RN Brown DM (1991) Isolation of a sequence common to A- and B-chromosomes of rye (*Secale cereale*) by microcloning. Plant Molecular Biology Reporter 9: 21–30. doi: 10.1007/BF02669286

[B42] Sanz MJ Loarce Y Ferrer E Fominaya A (2012) Use of tyramide-fluorescence in situ hybridization and chromosome microdissection for ascertaining homology relationships and chromosome linkage group associations in oats. Cytogenetic and Genome Research 136: 145–156. doi: 10.1159/000335641 22285909 10.1159/000335641

[B43] Schmidt T Heslop-Harrison JS (1998) Genomes, genes and junk: The large-scale organization of plant chromosomes. Trends in Plant Science 3: 195–199. doi: 10.1016/S1360-1385(98)01223-0

[B44] Schubert I Fransz PF Fuchs J de Jong JH (2001) Chromosome painting in plants. In: Sharma AK Sharma A (Eds) Chromosome Painting.Springer, Netherlands, 57–69. doi: 10.1007/978-94-010-0330-8_7 11741144

[B45] Shibata F Hizume M Kuroki Y (1999) Chromosome painting of Y chromosomes and isolation of a Y chromosome-specific repetitive sequence in the dioecious plant *Rumex acetosa*. Chromosoma 108: 266–270. doi: 10.1007/s004120050377 10460415 10.1007/s004120050377

[B46] Steflova P Hobza R Vyskot B Kejnovsky E (2013) Strong Accumulation of Chloroplast DNA in the Y Chromosomes of *Rumex acetosa* and *Silene latifolia*. Cytogenetic and Genome Research 142: 59–65. doi: 10.1159/000355212 24051898 10.1159/000355212

[B47] Stein N Ponelies N Musket T McMullen M Weber G (1998) Chromosome micro-dissection and region-specific libraries from pachytene chromosomes of maize (*Zea mays* L). Plant Journal 13: 281–289. doi: 10.1046/j.1365-313X.1998.00033.x

[B48] Tanurdzic M Banks JA (2004) Sex-determining mechanisms in land plants. Plant Cell 16: S61–S71. doi: 10.1105/tpc.016667 15084718 10.1105/tpc.016667 PMC2643385

[B49] Telenius H Carter NP Bebb CE Nordenskjold M Ponder BAJ Tunnacliffe A (1992) Degenerate oligonucleotide-primed PCR – general amplification of target DNA by a single degenerate primer. Genomics 13: 718–725. doi: 10.1016/0888-7543(92)90147-K 1639399 10.1016/0888-7543(92)90147-k

[B50] van Bakel H Stout JM Cote AG Tallon CM Sharpe AG Hughes TR Page JE (2012) The draft genome and transcriptome of *Cannabis sativa*. Genome Biology 12(10): R102. doi: 10.1186/gb-2011-12-10-r102 22014239 10.1186/gb-2011-12-10-r102 PMC3359589

[B51] Vyskot B Hobza R (2004) Gender in plants: sex chromosomes are emerging from the fog. Trends in Genetics 20: 432–438. doi: 10.1016/j.tig.2004.06.006 15313552 10.1016/j.tig.2004.06.006

[B52] Winge O (1929) On the nature of sex chromosome in *Humulus*. Hereditas 12: 53–63. doi: 10.1111/j.1601-5223.1929.tb02497.x

[B53] Yampolsky C Yampolsky H (1922) Distribution of sex forms in the phanerogamic flora. Bibliotheca Genetica 3: 1–62.

[B54] Zhang Y Zhang SG Qi LW Liu B Gao JM Chen CB Li XL Song WQ (2005) Construction of Poplar (*Populus tremula*) chromosome 1-specific DNA library by using a microdissection technique. Plant Molecular Biology Reporter 23: 129–138. doi: 10.1007/BF02772703

[B55] Zhong X-B de Jong JH Zabel P (1996) Preparation of tomato meiotic pachytene and mitotic metaphase chromosomes suitable for fluorescence *in situ* hybridization. Chromosome Research 4: 24–28. doi: 10.1007/BF02254940 8653264 10.1007/BF02254940

[B56] Zhou YH Hu ZM Dang BY Wang HA Deng XD Wang LL Chen ZH (1999) Microdissection and microcloning of rye (*Secale cereale* L.) chromosome 1R. Chromosoma 108: 250–255. doi: 10.1007/s004120050375 10460413 10.1007/s004120050375

[B57] Zonneveld BJM Leitch IJ Bennett MD (2005) First nuclear DNA amounts in more than 300 angiosperms. Annals of Botany 96: 229–244. doi: 10.1093/aob/mci170 15905300 10.1093/aob/mci170 PMC4246870

